# Deep distortions in faces and places

**DOI:** 10.3758/s13421-025-01805-x

**Published:** 2025-11-05

**Authors:** Daniel M. Bialer, C. J. Brainerd

**Affiliations:** 1 Department of Psychological Science, University of Arkansas, 216 Memorial Hall, Fayetteville, AR 72701 USA; 2 Department of Psychology and Human Neuroscience Institute, Cornell University, Ithaca, NY 14850 USA

**Keywords:** Deep distortions, Conjoint recognition, Fuzzy-trace theory, Verbatim and gist retrieval

## Abstract

Deep distortions occur when a person remembers multiple incompatible reality states as true, producing logical impossibilities. Those impossibilities include violations of basic logical principles such as additivity, countable additivity, and universal event. Previously, deep distortions have been detected in experiments using word lists and using real-world facts. We report two experiments in which we extend these findings to realistic pictures that figure prominently in legal cases. In Experiment 1, we presented participants with images of celebrity faces and asked them true or false questions about their identities, which were either conventional probes (e.g., “This is Alexander Hamilton”) or disjunctive probes (e.g., “This is Alexander Hamilton or James Madison”). In Experiment 2, we presented participants with images of locations around their local community, which were also paired with true or false probes about the identity of the locations that were either conventional probes (e.g., “This is Mann Library”) or disjunctive probes (e.g., “This is Uris Library or Mann Library”). In both experiments we found violations of additivity, countable additivity, and universal event. We also found that the incompatible identities of the faces and locations were not compensatory (i.e., as the acceptance rates for one incompatible identity increased, the acceptance rates for the other incompatible identities did not decrease). Additionally, we conducted conjoint recognition analyses and found that deep distortions were rooted in gist memory rather than verbatim memory. Overall, these finding suggest that deep distortions are also stimulated by realistic images that are highly relevant to legal cases.

There are sets of events whose members can never co-occur. For example, an airplane cannot simultaneously land at John F. Kennedy, LaGuardia, and Newark Liberty International Airports. Similarly, a person cannot be born on June 5, August 5, and October 5. If one considers all the days of the year, as the probability that a baby is born on a certain day increases, the sum of the probabilities that the baby is born on the other days of the year decreases, a rule known as compensation. Compensation causes incompatible events of this sort to follow certain logical constraints. For example, the sum of the probability that a baby is born on June 5 and the probability that a baby is born on August 5 must equal the probability that a baby is born on either June 5 or August 5, a principle known as additivity. This principle holds no matter how many days of the year are included. Therefore, it similarly holds that the sum of the probability that a baby is born on June 5, the probability that a baby is born on August 5, and the probability that a baby is born on October 5, must equal the probability that a baby is born on June 5 or August 5 or October 5, a principle known as countable additivity. Another logical axiom that must be obeyed by mutually exclusive events is universal event. This principle dictates that the sum of the probabilities of a subset of mutually exclusive events must be less than or equal to 1, and that the sum of the probabilities of all the mutually exclusive events must be equal to 1 (see Table 1 for examples).

**Table 1:** Examples of logical constraints on mutually exclusive events

Constraint	Example of baby’s date of birth
Additivity	*p*(June 5) + *p*(August 5) = *p*(June 5 V August 5)
	*p*(June 5) + *p*(October 5) = *p*(June 5 V October 5)
	*p*(August 5) + *p*(October 5) = *p*(August 5 V October 5)
Countable additivity	*p*(June 5) + p(August 5) + *p*(October 5) = *p*(June 5 V August 5 V October 5)
Universal event	*p*(June 5) + *p*(August 5) ≤ 1
	*p*(June 5) + *p*(August 5) + *p*(October 5) ≤ 1
	*p*(January 1) + *p*(January 2) + *p*(January 3) + . . . *p*(December 31) = 1

Although these logical constraints must be satisfied by events in the real world, that does not guarantee that they will be satisfied by people’s judgments about and memories of events. Tversky and Koehler (1994) and Rottenstreich and Tversky (1997), for example, found that when participants were asked to rate the probabilities of mutually exclusive events, the sum of the probability ratings tended to be greater than the probability rating of the disjunction of the events. This is a violation of additivity. Logical constraints such as additivity, countable additivity, and universal event have also been found to be regularly violated in episodic memory experiments (see Brainerd, 2021, 2022, for reviews). Brainerd (2021) termed these logical violations “deep distortions,” to distinguish them from distortions in item and source memory, which were termed “surface distortions.”

Surface distortions refer to false memories of individual events, whereas deep distortions refer to the relations between memories for different events. More explicitly, a person may remember multiple incompatible reality states as being true. For instance, imagine that on a visit to a gas station on Main Street, you witness an armed robbery. There are three gas stations on Main Street, a Shell, a British Petroleum (BP), and an ExxonMobil. When a police officer asks you a few weeks later, “Did the robbery you observed occur at the BP station?,” you answer “yes.” After all, you witnessed an armed robbery at a gas station on Main Street and it could have plausibly been the BP station. Had the police officer instead asked you “Did the robbery you observed occur at the Shell station?,” however, you might have also said “yes.” In fact, you might have even said “yes” if the police officer had asked you “Did the robbery you observed occur at the ExxonMobil station?” It is not that you believe that the robbery occurred at multiple gas stations simultaneously. You know that you only observed a single robbery and that it is a logical impossibility for the robbery to have occurred at multiple gas stations simultaneously. Yet, based on the framing of the question you receive from the police officer, you report contradictory memories of the location of the robbery.

There is a substantial literature in which these deep distortions have been measured under controlled conditions—most of them concerned with memory for words in laboratory experiments and a few concerned with memory for real-world facts. In this article, we report some new experiments of the latter sort, which removed methodological limitations of prior work and focused on facts that supply evidence in criminal cases. Before reporting our experiments, we provide some brief background on the two streams of deep distortion research, emphasizing key features of their methodologies as well as their findings.

## Background

### Deep distortions in word lists

In the above example, it is easy to see the presence of deep distortions. As a witness, you ascribed the crime to multiple incompatible locations in violation of basic logic. However, this example also highlights the major challenge in detecting deep distortions. The police officer only asked you whether the crime occurred at the BP station. The officer did not also ask you whether the location was the Shell station or ask you whether the location was the ExxonMobil station because your answer to one question would affect your answer to the others (i.e., answering “yes” to the BP station question may lead you to answer “no” to the Shell and ExxonMobil questions). This same limitation is present in standard false memory experiments. Typically, in a recognition experiment, participants are asked whether each item is an old, previously presented item. They are not asked about other mutually exclusive reality states, preventing detection of deep distortions.

The conjoint recognition paradigm, however, allows deep distortions to be measured in recognition experiments. In conjoint recognition, participants study a series of items and are then provided with a recognition test. Like typical false memory experiments, participants are tested with a mix of studied items (targets), related items (RDs), and unrelated items (UDs). Unlike typical false memory experiments, participants are also tested with varied recognition probes. Some are asked to accept targets and reject RDs and UDs (verbatim condition), some are asked to accept RDs and reject targets and UDs (gist condition), and some are asked to accept both targets and RDs and reject UDs (verbatim + gist condition). Conjoint recognition has been widely used (e.g., Abadie & Camos, 2019; Lampinen et al., 2006; Obidziński, 2021; Singer & Remillard, 2008; Stahl & Klauer, 2008) because it allows for the measurement of dual retrieval processes in false memory (Brainerd et al., 1999, 2001; for a review, see Brainerd et al., 2022).

In a review of the literature, Brainerd (2021) found that deep distortions were routinely detected in conjoint recognition experiments. To illustrate, consider the earliest deep distortion study, Brainerd et al. (2010). In two experiments, the authors presented participants with a series of Deese–Roediger–McDermott (DRM; Deese, 1959; Roediger & McDermott, 1995) lists. A DRM list consists of words, which are forward associates of a word known as a critical distractor (CD). After studying the DRM lists, participants completed two recognition tests, one immediately after the study phase and a second one a week later. The test items included targets, CDs, RDs, and UDs. Logically, the acceptance rates for any of the four types of test items should exhibit a pattern in which the sum of the acceptance rates in the verbatim and gist conditions equals the acceptance rate in the verbatim + gist condition [*p*(V) + *p*(G) = *p*(V + G)]. Instead, subadditive relations were found for targets, CDs, and RDs in both experiments. That is, the sum of the acceptance rates in the verbatim and gist conditions was significantly greater than the acceptance rate in the verbatim + gist condition [*p*(V) + *p*(G) > *p*(V + G)]. In a third experiment, Brainerd et al. also found that subadditive relations were present even when the words in the study list were unrelated. Further research has found that sub-additive relations are common in false memory. Brainerd (2021) examined a corpus of 373 recognition studies and found that there was strong evidence of violations of additivity. These studies suggest that deep distortions are commonly found in standard laboratory studies of episodic memory.

### Deep distortions in everyday memories

While deep distortions have been well-documented in laboratory studies, their presence in everyday memory is not well documented. Therefore, Brainerd et al. (2024, 2025) conducted a series of studies in which the to-be-remembered events were real-world facts. Specifically, they developed a distortion in everyday memory (DEM) instrument, which was a set of true or false memory probes about every day facts of the sort that are topics of *Jeopardy!* questions. The DEM consists of 100 true or false probes about various domains, including history, popular culture, music, science, sports, and geography. For each question, participants received one of six versions. First, participants could receive a true fact (*A* probes). Second, participants could receive one of two false facts (*B* probes and *C* probes). Third, participants could receive one of two disjunctions containing both the true fact and one of the false facts (*A* or *B* probes and *C* or *A* probes). Finally, participants could receive a disjunction containing both false facts (*B* or *C* probes). Each of the memory probes could have more than three plausible versions, so therefore, we did not present participants with an exhaustive set of plausible possibilities (see Table 2 for example memory probes from the DEM).

**Table 2:** Examples of the DEM items

Item and probe pairings	
Item 1:	
Topic: Holidays	
	A? = In the United Kingdom, the day after Christmas is known as Boxing Day.
	B? = In the United Kingdom, the day after Christmas is known as Victoria Day.
	C? = In the United Kingdom, the day after Christmas is known as Orangeman’s Day.
	A or B? = In the United Kingdom, the day after Christmas is known as Boxing Day or Victoria Day.
	B or C? = In the United Kingdom, the day after Christmas is known as Victoria Day or Orangeman’s Day.
	C or A? = In the United Kingdom, the day after Christmas is known as Orangeman’s Day or Boxing Day.
Item 2:	
Topic: History	
	A? = The first United States president to face impeachment was Andrew Johnson.
	B? = The first United States president to face impeachment was Richard Nixon.
	C? = The first United States president to face impeachment was Bill Clinton.
	A or B? = The first United States president to face impeachment was Andrew Johnson or Richard Nixon.
	B or C? = The first United States president to face impeachment was Richard Nixon or Bill Clinton.
	C or A? = The first United States president to face impeachment was Bill Clinton or Andrew Johnson.
Item 3:	
Topic: Literature	
	A? = Hamlet is Shakespeare’s longest play.
	B? = Romeo and Juliet is Shakespeare’s longest play.
	C? = Macbeth is Shakespeare’s longest play.
	A or B? = Hamlet or Romeo and Juliet is Shakespeare’s longest play.
	B or C? = Romeo and Juliet or Macbeth is Shakespeare’s longest play.
	C or A? = Macbeth or Hamlet is Shakespeare’s longest play.

*A* probes are true fact statements. *B* probes are the first false, plausible alternative to *A* probes. *C* probes are the second false, plausible alternative to *A* probes. *A* or *B* probes are true disjunctive facts including *A* probes and *B* probes as disjuncts. *B* or *C* probes are false disjunctive fact statements including *B* probes and *C* probes as disjuncts. *C* or *A* probes are true disjunctive fact statements including *C* probes and *A* probes as disjuncts.

Presenting the various conventional and disjunctive DEM probes allows for the detection of deep distortions. For example, violations of additivity are found when the ratio of the sum of the acceptance rates for two conventional probes to the acceptance rate of the disjunction of those two conventional probes [e.g., *p*(A) + *p*(B)/*p*(A-or-B)] is not equal to 1. This relation is subadditive when the ratio is greater than 1 and superadditive when the ratio is less than 1. Violations of countable additivity are found when the ratio of the sum of all three conventional probes to an approximation of the disjunction of all three conventional probes {e.g., [*p*(A) + *p*(B) + *p*(C)]/[*p*(A-or-B) + *p*(B-or-C) − *p*(B)]} is not equal to 1. As with additivity, a ratio greater than 1 indicates subadditivity and a ratio less than 1 indicates superadditivity. Violations of universal set are found when the sum of the acceptance probabilities of multiple conventional probes [e.g., p(A) + p(B) + p(C)] is greater than or equal to 1. As the false probes in the DEM are not an exhaustive set of plausible options (Brainerd et al., 2024), even a sum equal to 1 violates the universal event principle.

Brainerd et al. (2024) found that participants exhibited violations of additivity, countable additivity, and universal event across four experiments. Specifically, responses were subadditive. These violations of logical axioms were found regardless of whether the population was college students or the general United States public and regardless of whether participants simply made the binary true or false choice or made confidence ratings as well. Brainerd et al. (2025) extended this work to older adults, finding that they exhibited the same violations of logical axioms. The authors reported that, against logic, acceptances of the *A*, *B*, and *C* probes were not complementary (i.e., accepting one of them did not decrease the probability of accepting the others). Overall, these experiments produced the first evidence that deep memory distortions are present in memory for real-world events.

### Theoretical explanations of deep distortions

The study of deep distortions grew out of fuzzy-trace theory (FTT), whose principles predict such violations. According to FTT, memories are stored in parallel as verbatim and gist traces, with verbatim traces supporting acceptance of targets and rejection of related distractors and gist traces supporting acceptance of both. If one applies that distinction to conjoint recognition methodology, it is easy to see that deep distortions fall out. Imagine that a person is presented with a target item in a recognition test. Under the V and V + G conditions, the person should accept the target, and under the G condition, the person should reject the target. However, if the person retrieves gist traces, that promotes acceptance of the target in all three conditions. While logically *p*(V) + *p*(G) = *p*(V + G), the overacceptance of the target item when retrieving gist leads to the pattern *p*(V) + *p*(G) > *p*(V + G). The same distortion occurs for RDs. Under the G condition and V + G condition, participants should accept the RD, and under the V condition, participants should reject the RD. However, if a person retrieves gist traces, it also promotes acceptance of the RD in all three conditions leading to the *p*(V) + *p*(G) > *p*(V + G) pattern. This is true regardless of whether the test items are words from laboratory experiments or general knowledge facts from everyday life. To illustrate the latter point, if people retrieve verbatim traces, they should accept the BP station as the location of the crime and reject other gas stations on Main Street. If, instead, a person retrieves gist traces, they may accept the BP, Shell, and ExxonMobil stations as the location of the crime because each of these locations fit the gist of a gas station on Main Street. To evaluate that account, Brainerd et al. (2024) fit conjoint recognition models to memory for everyday facts and found that deep distortions were tied to gist retrieval rather than verbatim retrieval.

An alternative explanation of deep distortions comes from signal-detection theory (SDT; Green & Swets, 1966). According to SDT, memory is unidimensional. In recognition experiments, SDT predicts that an item is accepted as old if evidence in favor of that item passes a certain threshold and as new if evidence does not pass that threshold (Dunn, 2004). To return to the gas station example, a person may accept the BP, Shell, and ExxonMobil stations as the location of the crime because each of these locations passes a certain memory threshold for the crime location and is therefore accepted leading to violations of logic. The problem with unidimensional memory explanations is that they predict that acceptance rates for probes that tap the same memory content such as targets and related distractors should be strongly correlated. Instead, however, targets and related distractors are dissociated (Brainerd & Gordon, 1994; Brainerd & Reyna, 1993). The same issue has been found in deep distortion research. SDT would predict strong correlations between the acceptance rates for *A* probes versus *B* and *C* probes, but Brainerd et al. (2024) found that these correlations are 0 over probes and weakly positive over participants. The correlations over probes are the correct test of dissociation because the weak correlations over participants can be produced by individual differences in memory ability. Overall, previous deep distortion research has been better predicted by dual-process rather than unidimensional theories.

## Overview of the current experiments

The present study was conducted to explore the boundary conditions of deep distortions. It has two main goals. The first was to extend the measurement of deep distortions in everyday memory from general knowledge facts to real-world objects that are of forensic relevance. The second was to conduct more sensitive tests of the role of verbatim memory in deep distortions in everyday memory. With respect to the first goal, owing to the importance of memory in legal evidence, a longstanding goal of false memory research has been to evaluate whether laboratory memory illusions also affect legal evidence (for a review, see Brainerd & Reyna, 2005). Here, we investigated deep distortions in people’s memory for famous faces and for physical locations. Of course, accurate facial memory is central to the reliability of a key type of legal evidence, eyewitness identification of suspects (e.g., Wells et al., 2020), and famous faces have been used to examine the effect of familiarity on eyewitness identification performance (e.g., De Jong et al., 2005; Ross et al., 2023). Memory for physical locations is also a key type of legal evidence. For example, the Federal Rules of Evidence, Rule 901 (2023) states that to admit evidence “the proponent must produce evidence sufficient to support a finding that the item is what the proponent claims it to be,” and a component of that authentication usually involves specifying the item’s physical location.

Turning to the other goal, to further examine the role of verbatim memory in deep distortions for everyday memory, prior studies have found that verbatim retrieval plays virtually no role, whereas it plays a significant role in laboratory studies with word lists (Brainerd et al., 2024). The theoretical question is whether this is a fundamental difference between laboratory and everyday manifestations of deep distortions. To address that question, we created optimal conditions for verbatim retrieval by administering stimuli that are known to facilitate it—namely, pictures. Many classic studies demonstrate that retrieval of verbatim details is far better with pictures than with verbal materials (Paivio & Csapo, 1973; Shepard, 1967; Standing et al., 1970). Hence, we measured deep distortions in memory for faces and locations with pictures, rather than with verbal descriptions.

## Experiment 1

### Method

#### Participants

The participants included 118 college students (94 women, 21 men, one other, two unreported) who participated in exchange for course credits. The mean age was 20.1 years (range: 18–23). We aimed for a sample approximately equal to the sample used in Experiment 1 of Brainerd et al. (2024). We deleted the data from one participant who answered less than 10% of the questions for any by-participant analyses. We conducted a sensitivity using G*Power 3.1 (Faul et al., 2007), which found that the sample size we used provided enough power (1 − β > .80) to detect a small sized (Cohen’s *d* = .26) effect in a single-sample *t* test. Participants were tested online using the Qualtrics survey platform.

#### Materials

The materials were 110 photographs and paintings portraying an individual’s face. Each face was displayed at a width of 200 pixels with a height set to maintain the image’s aspect ratio. 100 of the images were representations of well-known figures from various domains (e.g., actors, historical figures, musicians, athletes, politicians). Each photograph was paired with a probe about the identity of the figure in the image. There were six possible probes that could be paired with each image. First, an image could be paired with a true probe about the identity of the figure in the image (hereafter designated as “A”). Second, an image could be paired with one of two plausible false probes about the identity of the figure in the image (hereafter designated as “B” and “C”). Third, an image could be paired with a true disjunction (hereafter designated as “A or B” and “C or A”). Finally, an image could be paired with a false disjunction (hereafter designated as “B or C”). See Table 3 for examples of each of the six possible stimuli.

**Table 3:** Examples of the famous face stimuli used in Study 1

Item and probe pairings	
Item 1:	
	[Painting of Alexander Hamilton]
	A? = This is Alexander Hamilton.
	B? = This is James Madison.
	C? = This is John Adams.
	A or B? = This is Alexander Hamilton or James Madison.
	B or C? = This is James Madison or John Adams.
	C or A? = This is John Adams or Alexander Hamilton.
Item 2:	
	[Photograph of Sophie Turner]
	A? = This is Sophie Turner.
	B? = This is Emilia Clarke.
	C? = This is Natalie Dormer.
	A or B? = This is Sophie Turner or Emilia Clarke.
	B or C? = This is Emilia Clarke or Natalie Dormer.
	C or A? = This is Natalie Dormer or Sophie Turner.
Item 3:	
	[Photograph of Michelle Kwan]
	A? = This is Michelle Kwan.
	B? = This is Kristi Yamaguchi.
	C? = This is Mirai Nagasu.
	A or B? = This is Michelle Kwan or Kristi Yamaguchi.
	B or C? = This is Kristi Yamaguchi or Mirai Nagasu.
	C or A? = This is Mirai Nagasu or Michelle Kwan.

*A* probes identify the true identity of the face. *B* probes identify the first false, plausible alternative to *A* probes. *C* probes identify the second false, plausible alternative to *A* probes. *A* or *B* probes are true disjunctive statements including *A* probes and *B* probes as disjuncts. *B* or *C* probes are false disjunctive statements including *B* probes and *C* probes as disjuncts. *C* or *A* probes are true disjunctive statements including *C* probes and *A* probes as disjuncts.

The other 10 images were AI-generated images of faces, which were added to serve as a measure of bias. For the AI-generated faces there were, of course, no correct answers. Therefore, for the AI-generated faces, *A*, *B*, and *C* were each invented names that were not associated with any celebrity.

#### Procedure

The procedure was conducted using the Qualtrics survey platform. Participants began the study by completing an informed consent form. Participants then read instructions and completed a set of examples, which included both conventional and disjunctive probes. They were also told that they should not look up the answers and that they would only have 10 s to answer each question. Participants were then presented with each of the 110 face images in a random order. Each image appeared centered on the screen with one of the six corresponding probes appearing below it centered in black font. Below the probe two radio buttons appeared corresponding to the answers “True” and “False.” The page would advance to the next image after the participant selected one of the two choices or after 10 s had elapsed. The purpose of the 10-s time limit was to prevent participants from searching for answers online. Each participant received approximately equal amounts of each of the six probe types (19 apiece for two of the probe types and 18 apiece for the remaining four probe types). Participants were randomly assigned to one of six possible combinations of the different probe types. Regardless of which combination a participant was assigned to, the images appeared in a random order.

### Results

The central question is whether deep distortions were still observed when we switched to pictures of familiar faces. Therefore, we implemented the analyses used by Brainerd et al. (2024) to measure these illusions. Specifically, we computed measures of additivity, countable additivity, universal event, and compensation, which are detailed in the sections below. Before we proceed, it is important to confirm that the faces and identities were indeed part of the long-term memories of the participants. If they are, *p*(A) should be greater than *p*(B) and *p*(C). Similarly, *p*(B-or-C) should be less than *p*(A-or-B) and *p*(C-or-A). To test these relations, we conducted a one-way analysis of variance (ANOVA) of the acceptance rates for the three conventional probes and a one-way ANOVA for the acceptance rates of the three disjunctive probes. The first ANOVA produced a significant main effect, *F*(2, 198) = 55.97, *p* < .001, ηp^2^ = .36. A Bonferroni-corrected post hoc analysis revealed the expected pattern, *p*(A) > *p*(B) = *p*(C). There was a significant difference between *p*(A) and both *p*(B), *p* < .001 and *p*(C), *p* < .001, but there was no significant difference between *p*(B) and *p*(C), *p* = .64. The disjunctive ANOVA also produced a significant main effect, *F*(2, 198) = 35.63, *p* < .001, ηp^2^ = .36. A Bonferroni-corrected post hoc analysis revealed the expected pattern, *p*(A-or-B) = *p*(C-or-A) > *p*(B-or-C). There was a significant difference between *p*(B-or-C) and both *p*(A-or-B), *p* < .001 and *p*(C-or-A), *p* < .001, but there was not a significant difference between *p*(A-or-B) and *p*(C-or-A), *p* = 1. The acceptance probabilities for all six probes can be seen in Table 4. One can also see that the same patterns were not present in the AI-generated faces, for which the participants could not possibly have any long-term memory.

**Table 4:** Mean acceptance probabilities for each probe type in Experiment 1

Probe types	Stimulus types	
	Famous faces	AI-generated faces
Conventional statement probes:		
A	0.61 (0.19)	0.31 (0.12)
B	0.37 (0.17)	0.29 (0.09)
C	0.40 (0.17)	0.33 (0.14)
Disjunctive statement probes:		
A or B	0.62 (0.18)	0.38 (0.15)
B or C	0.46 (0.17)	0.40 (0.16)
C or A	0.63 (0.19)	0.40 (0.14)

For the famous faces, A, B, and C are, respectively, acceptance of the correct identity of the face, acceptance of the first incorrect identity, and acceptance of the second incorrect identity. For the AI-generated faces, A, B, and C are, respectively, acceptance of the first, second, and third incorrect identities of the face.

#### Additivity

To determine whether the data violated the logical principle of additivity, we examined whether three ratios in Table 5, [*p*(A) + *p*(B)]/*p*(A-or-B), [*p*(B) + *p*(C)]/*p*(B-or-C), and [*p*(A) + *p*(C)]/*p*(C-or-A), differed reliably from 1. If a ratio equals 1, the data are additive. (The data are subadditive if the ratio is >1 and superadditive if it is <1). To test whether the ratios differed from 1, we conducted one-sample *t* tests. We found that all three additivity ratios were significantly greater than 1, *t*(99) = 13.49, *p* < .001, *t*(99) = 12.12, *p* < .001, *t*(99) = 14.42, *p* < .001. See rows one, two, and three of Table 5 for the additivity ratio values. As can be seen, all three ratios produced subadditive relations, showing that participants connected the faces with more than one identity, a logical impossibility.

**Table 5:** Deep distortion measures for Experiment 1

Distortion measures	Stimulus types
	Famous faces
Additivity:	
[*p*(A) + *p*(B)]/*p*(A-or-B)	1.65
[*p*(B) + *p*(C)]/*p*(B-or-C)	1.82
[*p*(A) + *p*(C)]/*p*(C-or-A)	1.70
Countable additivity	
[*p*(A) + *p*(B) + *p*(C)]/[*p*(A-or-B) + *p*(B-or-C) − *p*(B)]	2.21
[*p*(A) + *p*(B) + *p*(C)]/[*p*(A-or-B) + *p*(C-or-A) − *p*(A)]	2.37
[*p*(A) + *p*(B) + *p*(C)]/[*p*(C-or-A) + *p*(B-or-C) − *p*(C)]	2.26
Universal set:	
* p*(A) + *p*(B) +* p*(C)	1.38
* p*(A) + *p*(B)	0.98
* p*(A) + *p*(C)	1.00

For the famous faces, A, B, and C are, respectively, acceptance of the correct identity of the face, acceptance of the first incorrect identity, and acceptance of the second incorrect identity.

#### Countable additivity

To test whether the data violated countable additivity, we examined whether three ratios, [*p*(A) + *p*(B) + *p*(C)]/[*p*(A-or-B) + *p*(B-or-C) − *p*(B)], [*p*(A) + *p*(B) + *p*(C)]/[*p*(A-or-B) + *p*(C-or-A) − *p*(A)], and [*p*(A) + *p*(B) + *p*(C)]/[*p*(C-or-A) + *p*(B-or-C) − *p*(C)], differed reliably from 1. If the ratio equals 1, the data satisfy countable additivity. To test whether the ratios differed from 1, we conducted one-sample *t* tests. We found that all three ratios were significantly greater than 1, *t*(99) = 11.40, *p* < .001, *t*(99) = 13.93, *p* < .001, *t*(99) = 11.63, *p* < .001. See the fourth, fifth, and six rows of Table 5 for the countable additivity ratio values. As can be seen, similar to additivity, all three ratios produced subadditive relations, showing that participants were connecting the faces with all three identities.

#### Universal event

To test whether the data violated the universal event principle, we examined whether three sums in Table 5, *p*(A) + *p*(B) +* p*(C), *p*(A) + *p*(B), and *p*(A) +* p*(C), differed reliably from 1. Here, we included only three plausible alternatives as to the identity of the famous faces. This, however, is a nonexhaustive set. For example, for the painting of Alexander Hamilton we included the alternatives of Alexander Hamilton, James Madison, or John Adams, but participants could also plausibly believe that the identity of the man in the painting is some other colonial figure, such as John Hancock, John Jay, or Samuel Adams. Logically, the sum of the acceptance probabilities of all plausible alternatives should equal 1. Therefore, the three sums we examined should logically be substantially less than 1 because we were far from providing an exhaustive set of plausible alternatives in any of our stimuli. We found that the sum of *p*(A) + *p*(B) +* p*(C) was significantly greater than 1, *t*(99) = 12.00, *p* < .001. The sums of *p*(A) + *p*(B) and *p*(A) + *p*(C) did not differ reliably from 1. These results indicate deep distortions, as universal event proscribes that these sums should be significantly below 1. See the seventh, eighth, and ninth rows of Table 5 for the universal event values. In row seven, we can see that the sum of *p*(A) + *p*(B) +* p*(C) was much greater than 1 (at 1.38).

#### Compensation

Logically, acceptance of the three incompatible candidates (A, B, and C) for an identity should be compensatory. Objectively, as the probability of one candidate goes up, the probability of the other candidates goes down. Therefore, if memory is obeying logical constraints, we should see compensation among the three candidates’ acceptance probabilities. To test this, we computed partial correlations for the probabilities of acceptance for the three candidates (see Table 6). We computed these correlations in two different ways. First, we ran the correlations by each individual famous face probe. Second, we ran the correlations over each participant. We found, again, that the participants did not adhere to logical constraints. While the partial correlations should be negative, the average correlation by probe was around 0 and the average correlation by participant was positive, at .37.

**Table 6:** Partial correlations between* A*, *B*, and *C* probes in Experiment 1

Measurement level	
By probe:	
* r*_AB_	−.02
* r*_AC_	−.09
* r*_BC_	.27*
*M*	.05
By participant:	
* r*_AB_	.19*
* r*_AC_	.27*
* r*_BC_	.67**
* M*	.37*

For the famous faces, A, B, and C are, respectively, acceptance of the correct identity of the face, acceptance of the first incorrect identity, and acceptance of the second incorrect identity.

*p < .05, **p < .001.

## Discussion

Although Brainerd et al. (2024) uncovered deep distortions in everyday memory, the boundary conditions of their presence have not yet been determined. As FTT predicted and Brainerd et al. supported that deep distortions result from reliance upon gist traces, we sought to examine whether deep distortions would be present for everyday memories of visual stimuli, which have typically induced greater reliance upon verbatim traces. Specifically, we chose to examine the presence of deep distortions in the identification of well-known figures. This allowed us to examine deep distortions in a context directly relevant in an applied setting—namely, in eyewitness identification.

We presented participants with a series of images of celebrity faces paired with conventional and disjunctive statement probes identifying the subject of the image. This permitted comparisons of acceptance rates for conventional and disjunctive probes to detect violations of additivity and countable additivity. Consistent with Brainerd et al. (2024), we found clear evidence of violations of both. We also examined violations of universal event and found that summing together acceptance rates for the correct conventional probe (A) and one or both incorrect conventional probes (B and C) produced sums that were statistically greater than or equal to 1. This again, reveals deep distortions. We also examined whether the acceptance rates for the different conventional probes were compensatory. Logically, as the acceptance rate of one alternative increases the acceptance rates for the others should decrease. However, we found that the partial correlations between the acceptance rates for pairs of incompatible probes were either positive or nonsignificant. Overall, we found clear evidence of deep distortions in materials besides the DEM. In the General Discussion, we further consider the roles of verbatim and gist in deep distortions.

## Experiment 2

In Experiment 1, we determined that deep distortions were present in identification of visual materials. However, this was the first experiment to detect deep distortions using such materials. Therefore, we conducted Experiment 2 to examine whether we could detect deep distortions in another set of visual materials that are relevant in legal contexts, photographs of locations.

### Method

#### Participants

The participants included 120 students (81 women, 34 men, one other, four unreported) who participated in exchange for course credits. All the participants attended college on the same campus. The mean age was 20.0 years (range: 18–28). We aimed for a sample approximately equal to the sample used in Experiment 1 of Brainerd et al. (2024). We conducted a sensitivity using G*Power 3.1 (Faul et al., 2007), which found that the sample size we used provided enough power (1 − β > .80) to detect a small sized (Cohen’s *d* = .26) effect in a single-sample *t* test. Participants were tested online using the Qualtrics survey platform.

#### Materials

The materials were 98 photographs portraying a location in or around the campus where the participants were attending college. Each photograph was displayed at a width of 266 pixels and height of 200 pixels. Similar to Experiment 1, the photographs were each paired with a probe about the identity of the location. The six types of probes were also the same as those in Experiment 1. See Table 7 for examples of each of the six possible stimuli. Only five of the six types of probes were displayed for one of the photographs, so we eliminated those questions for a final total of 97 photographs.

**Table 7:** Examples of the location stimuli used in Study 2

Items and probe pairings	
Item 1:	
	[Photograph of Warren Hall]
	A? = This is Warren Hall.
	B? = This is Rockefeller Hall.
	C? = This is Tjaden Hall.
	A or B? = This is Warren Hall or Rockefeller Hall.
	B or C? = This is Rockefeller Hall or Tjaden Hall.
	C or A? = This is Tjaden Hall or Warren Hall.
Item 2:	
	[Photograph of Mann Library]
	A? = This is Mann Library.
	B? = This is Adelson Library.
	C? = This is Uris Library.
	A or B? = This is Mann Library or Adelson Library.
	B or C? = This is Adelson Library or Uris Library.
	C or A? = This is Uris Library or Mann Library.
Item 1:	
	[Photograph of Taughannock Falls]
	A? = This is Taughannock Falls.
	B? = This is Horseshoe Falls.
	C? = This is Buttermilk State Park.
	A or B? = This is Taughannock Falls or Horseshoe Falls.
	B or C? = This is Horseshoe Falls or Buttermilk State Park.
	C or A? = This is Buttermilk State Park or Horseshoe Falls.

*A* probes identify the true identity of the location. *B* probes identify the first false, plausible alternative to *A* probes. *C* probes identify the second false, plausible alternative to *A* probes. *A* or *B* probes are true disjunctive statements including *A* probes and *B* probes as disjuncts. *B* or *C* probes are false disjunctive statements including *B* probes and *C* probes as disjuncts. *C* or *A* probes are true disjunctive statements including* C* probes and *A* probes as disjuncts.

#### Procedure

The procedure was nearly identical to that of Experiment 1. As in Experiment 1, participants had 10 s to respond “True” or “False” to a series of images that were paired with one of the six probe types. Each participant also received approximately equal amounts of each of the six probe types (17 apiece for two of the probe types and 16 apiece for the remaining four probe types).

### Results

As in Experiment 1, we computed measures of additivity, countable additivity, universal event, and compensation. For Experiment 2, we also examined whether *p*(A) was greater than *p*(B) and *p*(C) and whether *p*(B-or-C) was less than *p*(A-or-B) and *p*(C-or-A) to confirm that the names of the locations were in the participants’ long-term memories. We ran a one-way ANOVA for the conventional probes and a second one-way ANOVA for the disjunctive probes. The conventional ANOVA produced a significant main effect, *F*(2, 192) = 132.12, *p* < .001, ηp^2^ = .58. Bonferroni corrected post hoc analysis revealed the expected pattern, *p*(A) > *p*(B) = *p*(C). There was a significant difference between *p*(A) and both *p*(B), *p* < .001 and *p*(C), *p* < .001, but there was no significant difference between *p*(B) and *p*(C), *p* = .083. The disjunctive ANOVA also produced a significant main effect, *F*(2, 192) = 93.45, *p* < .001, ηp^2^ = .49. Bonferroni-corrected post hoc analysis revealed the expected pattern, *p*(A-or-B) = *p*(C-or-A) > *p*(B-or-C). There was a significant difference between *p*(B-or-C) and both *p*(A-or-B), *p* < .001 and *p*(C-or-A), *p* < .001, but there was no significant difference between *p*(A-or-B) and *p*(C-or-A), *p* = .76. The acceptance probabilities for all six probes can be seen in Table 8.

**Table 8:** Mean acceptance probabilities for each probe type in Experiment 2

Probe types	Stimulus types
	Locations
Conventional statement probes:	
A	0.71 (0.21)
B	0.33 (0.21)
C	0.28 (0.20)
Disjunctive statement probes:	
A or B	0.74 (0.19)
B or C	0.43 (0.21)
C or A	0.72 (0.20)

For the locations, A, B, and C are, respectively, acceptance of the correct name of the location, acceptance of the first incorrect name, and acceptance of the second incorrect name.

#### Additivity

To test whether the data violated additivity, we examined whether the additivity ratios in Table 9, [*p*(A) + *p*(B)]/*p*(A-or-B), [*p*(B) + *p*(C)]/*p*(B-or-C), and [*p*(A) + *p*(C)]/*p*(C-or-A), differed reliably from 1. We computed one-sample *t* tests for each additivity ratio. We found that all three additivity ratios were significantly greater than 1, *t*(96) = 10.53, *p* < .001, *t*(96) = 6.21, *p* < .001, *t*(96) = 7.47, *p* < .001. See rows one, two, and three of Table 9 for the additivity ratio values. Similar to Experiment 1, all three ratios produced subadditive relations, showing that participants connected the pictures with incompatible locations.

**Table 9:** Deep distortion measures for Experiment 2

Distortion measures	Stimulus type
	Locations
Additivity:	
[*p*(A) + *p*(B)]/*p*(A-or-B)	1.46
[*p*(B) + *p*(C)]/*p*(B-or- C)	1.53
[*p*(A) + *p*(C)]/*p*(C-or-A)	1.46
Countable additivity	
[*p*(A) + *p*(B) + *p*(C)]/[*p*(A-or-B) + *p*(B-or-C) - *p*(B)]	1.73
[*p*(A) + *p*(B) + *p*(C)]/[*p*(A-or-B) + *p*(C-or-A) - *p*(A)]	1.93
[*p*(A) + *p*(B) + *p*(C)]/[*p*(C-or-A) + *p*(B-or-C) - *p*(C)]	1.62
Universal set:	
* p*(A) + *p*(B) +* p*(C)	1.32
* p*(A) + *p*(B)	1.05
* p*(A) + *p*(C)	0.99

For the locations, A, B, and C are, respectively, acceptance of the correct name of the location, acceptance of the first incorrect name, and acceptance of the second incorrect name.

#### Countable additivity

To test whether the data violated countable additivity, we examined whether the ratios in Table 9, [*p*(A) + *p*(B) + *p*(C)]/[*p*(A-or-B) + *p*(B-or-C) - *p*(B)], [*p*(A) + *p*(B) + *p*(C)]/[*p*(A-or-B) + *p*(C-or-A) - *p*(A)], and [*p*(A) + *p*(B) + *p*(C)]/[*p*(C-or-A) + *p*(B-or-C) - *p*(C)], differed reliably from 1. We computed three one-sample *t* tests. We found that all three additivity ratios were significantly greater than 1, *t*(96) = 8.53, *p* < .001, *t*(96) = 11.24, *p* < .001, *t*(96) = 9.73, *p* < .001. See the fourth, fifth, and six rows of Table 9 for the countable additivity ratio values. As in Experiment 1, all three ratios produced sub-additive relationships, showing that the pictures were connected to all three incompatible locations.

#### Universal event

To test whether the data violated the universal event principle, we examined whether the sums in Table 9, *p*(A) + *p*(B) +* p*(C), *p*(A) + *p*(B), and *p*(A) +* p*(C), differed reliably from 1. As in Experiment 1, we found that the sum of *p*(A) + *p*(B) +* p*(C) was significantly greater than 1, *t*(96) = 8.84, *p* < .001, but that the other two sums did not differ from 1. Importantly, the other two were not significantly less than 1, which is what universal event requires for both sums. See the seventh, eighth, and ninth rows of Table 9 for the universal event values. The value for *p*(A) + *p*(B) +* p*(C) was 1.32, well over what is logically possible.

#### Compensation

To test compensation, we computed partial correlations among the probabilities of acceptance for the three candidates (see Table 10). We computed these correlations by each individual location probe and over each participant. The patterns we found were largely consistent with Experiment 1. While the partial correlations should be strongly negative, the average correlation by probe was around 0 and the average correlation by participant was positive, at .26.

**Table 10:** Partial correlations between A, B, and C probes in Experiment 2

Measurement Level	
By Probe:	
* r*_AB_	-.07
* r*_AC_	-.10
* r*_BC_	.27*
* M*	.03
By Participant:	
* r*_AB_	.23*
* r*_AC_	-.04
* r*_BC_	.59**
* M*	.26*

For the locations, A, B, and C are respectively acceptance of the correct name of the location, acceptance of the first incorrect name of the location, and acceptance of the second incorrect name of the location. *p < .05, **p < .001.

## Discussion

In Experiment 1, we found that deep distortions could be detected in everyday memory for visual stimuli. We found that deep distortions were present when identifying celebrity faces. The purpose of Experiment 2 was to examine whether we could replicate these findings in another forensically relevant context. We presented participants with a series of familiar locations around their college with conventional and disjunctive statement probes identifying the subject of the image. Like Experiment 1, we found violations of additivity, countable additivity, and universal event. Similarly, we found that acceptance rates for the three conventional probes were not compensatory. These results suggest that deep distortions can be detected in everyday memory for varied visual materials. In the General Discussion we consider the theoretical rationale for these findings as well as applications of this research to forensic settings.

## General discussion

In some recent experiments, Brainerd et al. (2024) found that the same deep distortions that had been repeatedly observed in laboratory episodic memory experiments are also observed in everyday memory for real-word facts. As the latter results were predicted on theoretical grounds, we tested the boundary conditions of that prediction by determining whether the same distortions occur when real-world facts are encoded in a very different format—namely, as pictures. We also focused on facts that are of prime importance in legal cases: people’s faces and physical locations. Typically, pictures make gist retrieval more difficult, as compared with the words and sentences that have figured in prior deep distortion studies. As FTT posits that gist retrieval is responsible for deep distortions, it was possible that deep distortions would disappear or be greatly reduced by pictorial encoding. Our results, however, were remarkably similar to those of studies in which these illusions were measured with words and sentences. Specifically, the sum of the acceptance probabilities of two incompatible reality states was greater than the acceptance probability of the disjunction of the two reality states (violating additivity), the sum of the acceptance probabilities of three incompatible reality states was greater than the acceptance probability of the disjunction of the three reality states (violating countable additivity), the sum of the acceptance of three incompatible reality states was greater than 1 (violating universal event), and the acceptance probabilities of incompatible memories were not negatively correlated (violating compensation).

Readers may notice that the deep distortions that we report are only detected at the item level or at the participant level, but not at the item × participant level (i.e., an individual participant is not reporting multiple incompatible reality states in these studies). The reason is that it is nearly impossible to collect data from the same participant for multiple probes of the same item without introducing repeated testing confounds. Therefore, we measured deep distortions between participant for each item and within participant across items. While these methods are imperfect, we believe that item × participant deep distortions are present. As participants were randomly assigned to groups that determined which probes they received and the experiments were sufficiently powered, we can reasonably assume that each group had an approximately equal knowledge base. If the framing of the probes did not affect participant answers, we would not expect to detect deep distortions at the item and participant levels, especially so robustly. There is also precedent for using between-participant designs to detect logical violations. The use of between-subjects measures, for example, has been employed in previous deep distortion studies (Brainerd et al., 2024, 2025), as well as classic studies of support theory (e.g., Rottenstreich & Tversky, 1997; Tversky & Koehler, 1994). It is, however, important to reiterate that these studies as well as the current study did not measure at the item × participant level, so it is impossible for us to determine whether participants are remembering multiple incompatible reality states as true.

One distinction between our study and some previous studies using pictorial stimuli is that we are asking participants to retrieve verbal information that connects with the picture rather than the visual information itself. In contrast, Shepard (1967) and Standing et al. (1970) asked participants to identify exact pictures in a recognition test. While Paivio and Csapo (1973) did request verbal descriptions of studied images, the descriptions were for images that were no longer being presented, so this task required retrieval of the studied image. In contrast, our study asks participants to retrieve verbal information about an image. Overall, however, the indicated conclusion is that deep distortions affect people’s memories across very different encoding formats, and they affect people’s memories for faces and places. In the remainder of this section we consider some of the theoretical and forensic implications of our findings.

## Theoretical implications

Our results are consistent with previous research that supports FTT’s explanation of deep distortions over a unidimensional explanation. Specifically, we found that the acceptance rates for A probes did not correlate with B probes or C probes when we examined the partial correlations by probe and the probes were weakly correlated when we examined the partial correlations by participant. Now, we connect our results more directly to FTT’s theoretical analysis of deep distortions. As detailed earlier, FTT predicted deep distortions in episodic and semantic memory as by-products of gist retrieval. If that analysis is correct, the participants in our experiments must have been relying on gist processing much of the time, as we detected quite strong deep distortions. To test this, we measured the actual amounts of gist retrieval in our experiments by fitting a conjoint-recognition model to the data. Conjoint-recognition models have been used in various memory and reasoning experiments to disentangle the contributions of gist retrieval, verbatim retrieval, and response bias to performance (for a review, see Brainerd et al., 2022). The conjoint recognition model for real-world fact memory measures the effects of these retrieval processes on responses to the six types of memory probes that were administered in our experiments (see Table 10). The model contains a total of five parameters. V_A_ is the probability that a participant will accept *A*, *A*-or-*B*, and *C*-or-*A* probes and reject *B*, *C*, and *B*-or-*C* probes based on retrieval of verbatim traces of learning the target fact (e.g., the face of a famous person in Experiment 1). If verbatim retrieval fails, G_A_ is the probability that a participant will accept *A*, *A*-or-*B*, and *C*-or-*A* probes based on retrieval of gist traces of the target fact. If verbatim retrieval fails, G_B_ is the probability that a participant will accept *B*, *A*-or-*B*, and *B*-or-*C* probes based on retrieval of gist traces of the target fact. If verbatim retrieval fails, G_C_ is the probability that a participant will accept *C*, *B*-or-*C*, and *C*-or-*A* probes based on retrieval of gist traces of the target fact. Finally, if verbatim and gist retrieval both fail, *b* is the probability of accepting any of the six probes via nonmemorial response bias. Mathematical details of this model and statistical methods of applying it to data may be found in Brainerd et al. (2024, Appendix).

We found that this model fit the data of both our experiments well. The model fit test is a G^2^(1) statistic, where a value of 3.84 or higher is the critical value to reject the null hypothesis that the model fits the data. The G^2^ values were far below this critical value, indicating excellent fit (see Table 11). When we estimated the model’s five parameters, the results confirmed previous findings that deep distortions are gist-based. First, the estimates of V_A_ were always 0, indicating no verbatim memory for these faces and places. Second, G_A_, G_B_, and G_C_ were all substantially greater than 0 in both experiments, indicating that participants were relying upon gist retrieval to make their judgments. G_A_ values were, in particular, quite large (.40 in Experiment 1 and .62 in Experiment 2). This suggests that approximately half of the acceptances of correct memory probes were gist-based. Another salient pattern in our data is that the G_A_ values were consistently larger than the G_B_ and G_C_ values. This pattern is consistent with previous conjoint recognition findings that parameters for gist retrieval are greater for old items than for new, but related items (Brainerd et al., 2022). The pattern is also consistent with the previous estimates of these parameters in answers to probes for general knowledge facts (Brainerd et al., 2024).

A possible explanation for these parameter results is that the gist of false but similar items does not perfectly match the gist of true items. Although we attempted to match the gist of false and correct facts, it is inevitable that the match will be imperfect. For example, in Experiment 1, one of the stimuli we presented was a photograph of Lisa Kudrow. If participants are relying on the gist of actresses from the television show *Friends*, they may accept that the photograph is of Courtney Cox, another actress from the show. If instead, participants are relying on the gist of blonde actresses, that would not support acceptance of Courtney Cox, a famously brunette actress, and therefore, this prompt would be rejected when paired with the blonde image of Lisa Kudrow. Overall, the modeling results follow the same pattern observed by Brainerd et al. (2024)—that deep distortions are driven by gist retrieval.

Experiment 2 provided an opportunity to conduct an additional test for the presence of verbatim retrieval in our memory tasks. We were able to approximate participants’ levels of experience with the physical locations that were tested. Because the participants were undergraduates at a four-year university in a small college town and the tested locations were campus and community locations, experience with those locations was positively correlated with number of semesters on campus. We re-fit the model to the data of two subsets of participants—namely, participants who had spent at least seven semesters on campus and participants who spent six or fewer semesters on campus. Presumably, the participants who spent seven or more semesters on campus should have stronger verbatim memories of the locations than the other participants because they have had more opportunities to form the association between the location and its name through their daily interactions with their environment. However, the conjoint-recognition analysis showed that the values of V_A_ were still 0 suggesting that even after seven semesters, participants were not vividly retrieving the instances in which they made or reinforced the association between the location and its name. In fact, the salient difference between the two subsets was in the G_A_ parameter: G_A_ values were larger for participants who had spent seven or more semesters on campus, leading to some improvements on the task. However, those participants still exhibited robust deep distortions. See Table 11 for the parameter estimates for this subset of participants in Experiment 2. Overall, at the level of retrieval processes, our results are consistent with both FTT’s predictions about deep distortions and with prior experiments that relied on purely verbal instruments.

**Table 11:** Parameter estimates and goodness-of-fit values for the conjoint recognition model

Experiment	Statistics	G^2^	V_A_	G_A_	G_B_	G_C_	*b*
Unrestricted model							
Experiment 1		0.24	0	0.40	0.08	0.12	0.33
Experiment 2							
All data		0.32	0	0.62	0.19	0.12	0.18
1–6 semesters		0.21	0	0.58	0.17	0.11	0.21
Only 7+ semesters		0.95	0	0.75	0.22	0.12	0.08

## Applied implications

The key advance in our understanding of deep distortions lies in the fact that, consistent with theoretical prediction, we now know that they occur for both of the main formats in which people encode items in memory experiments. Beyond that, we measured deep distortions with images that figure routinely in legal evidence. For instance, facial images like those that were administered in Experiment 1 are the backbone of eyewitness identifications, which isolate suspects during criminal investigations and provide evidence of guilt in sworn testimony. Further, images of locations like those in Experiment 2 are admitted and authenticated in sworn testimony during court proceedings. Hence, our results also generate some hypotheses about memory distortions in legal evidence.

With respect to the facial images in Experiment 1, our procedure was similar to one of two major methods of administering eyewitness identifications: showups (e.g., Colloff & Wixted, 2020; Gonzalez et al., 1993; Gronlund et al., 2012). Our results illustrate the critical importance of the content of memory probes in such identifications: We found that participants’ judgments about the identity of faces depended more on the content of the memory probe than on the actual identity of the faces; otherwise, deep distortions would have been absent. Here, a finding of fundamental significance is that participants ascribed contradictory identities to the same face.

These results are consistent with recent best-practice protocols, which note the high error rates that have been reported in studies of showups and recommend against their use in criminal investigations except in specific circumstances (e.g., Wells et al., 2020). The likely reason for showups’ high error rates is that they allow witnesses to rely more on gist retrieval than the other major identification procedure, lineups (Wixted & Wells, 2017). As we saw in Experiment 1, relying upon gist retrieval leads to multiple memory distortions, which are inherently illogical. Forensically, these distortions converge on heightened false positive rates: The chances that innocent people will be identified as culprits are obviously greater if witnesses rely on the gist of culprits’ faces (e.g., young, thin, male, Hispanic) than if they rely on literal verbatim memories.

We should add that participants’ tendency to rely on gist retrieval in showups can be combatted in various ways. One method is to encourage eyewitnesses to use stronger remembering criteria to make positive identifications only when they vividly recollect culprits’ faces (Brainerd & Reyna, 2005). For example, some lineup studies have included instructions that discourage participants from making an identification unless they are 100% sure of their selection (Meissner et al., 2005; Mickes et al., 2017). A related method is to instruct witnesses that they have the option of simply not reporting anything in response to a showup (e.g., Koriat & Goldsmith, 1996). However, the simplest solution is to administer lineups rather than showups. In lineups, where the gist of all the faces is the same, relying on gist traces does not discriminate among the faces, encouraging reliance on verbatim retrieval (Wixted & Wells, 2017). Switching to lineups, however, will not completely eliminate reliance on gist. Therefore, our results also underscore the importance of evidence-based suspicion before conducting lineups. The idea of evidence-based suspicion is that a lineup should not be conducted unless there is a sufficient level of evidence supporting the guilt of the suspect (see Well et al., 2020). In essence, we are suggesting that increasing the percentage of *A* probes presented to eyewitnesses and decreasing the percentage of *B* and *C* probes will result in fewer wrongful identifications of innocent suspects.

The results of Experiment 2 have parallel implications for the accuracy of witnesses’ identification of locations that figure in legal evidence, such as crime scenes. Federal Rules of Evidence, Rule 901 (2023) requires that the proponent of an item of evidence (including a photograph) “must produce evidence sufficient to support a finding that the item is what the proponent claims it is” (Fed R. Evid. 901(a)). One permitted source of evidence is “testimony that an item is what it is claimed to be” from a witness with knowledge (Fed. R. Evid. 901(b)(1)). This rule applies to all federal courts, and the principles outlined by Rule 901 are recognized in most state courts. When the item is a physical location, the testimony that “an item is what it is claimed to be” is usually produced by witness identification of a photograph. For example, consider *Commonwealth v. Davis*, a Massachusetts state court case, in which a detective who had visited and recorded the scene of a shooting and car crash authenticated photographs of the crime scene by testifying that the photographs “were fair and accurate representations of the scene of the shooting and crash” (Commonwealth v. Davis, 2020, Section 2(a), paragraph 7).

The implication of the results of Experiment 2 suggest that such photographic authentications of locations will be subject to the same memory distortions as facial identifications. According to our results, people ascribed incorrect and mutually contradictory identities to locations in a university community even when they had spent over three years in the community. Similar to facial identification, such errors depended more on the content of memory probes than on the actual accuracy of the photographs. This suggests that if witnesses are asked whether a photograph of a location is the location where a crime occurred, they will falsely assent to photographs that do not match the location where the crime scene occurred, and they will assent to mutually contradictory photographs. Overall, our results demonstrate that people are susceptible to remembering gist-consistent photographs of locations, regardless of whether they are correct or incorrect, and that these memories are non-compensatory. When memory for physical locations is measured through photographic authentication, people’s memories are subject to the same logical distortions as eyewitness identification of suspects via showups.

## Concluding comments

Our results add to a growing literature on deep distortions, a new form of false memory that is concerned with illogical relations among memories of different facts, rather than false memories of specific facts. Unlike traditional false-memory research, which is focused on episodic memory, the study of deep distortions spans episodic and semantic memory. We now know that robust deep distortions occur when memory is measured in pictorial as well as verbal formats, a method that normally reduces reliance on gist retrieval. Perhaps more important, our results show that deep distortions occur for typical pictorial representations of two types of facts that figure centrally in legal evidence: people’s faces and physical locations. In that connection, when witnesses make identifications with showups or authenticate photographs of locations, their memories will be distorted in ways that violate basic logical rules. Our results show that people will remember multiple incompatible identities and physical locations for the same image, just as they do with words in laboratory experiments.

## Data Availability

Organized data are included on OSF (https://osf.io/kq9jc/). Materials are available upon request with the corresponding author. Neither of the experiments was preregistered.

## Code Availability

Code is available upon request with the corresponding author.

## Declarations

### Author note

The research reported in this article was supported by United States Department of Agriculture Grant NYC-321444.
